# Multiple Endocrine Neoplasia Type 1 (MEN1): An Update and the Significance of Early Genetic and Clinical Diagnosis

**DOI:** 10.3389/fendo.2019.00339

**Published:** 2019-06-11

**Authors:** Crystal D. C. Kamilaris, Constantine A. Stratakis

**Affiliations:** Section on Endocrinology and Genetics and Endocrinology Inter-institute Training Program, Eunice Kennedy Shriver National Institute of Child Health and Human Development, National Institutes of Health, Bethesda, MD, United States

**Keywords:** MEN1, hyperparathyroidism, pituitary adenoma, enteropancreatic tumor, carcinoid, adrenocortical tumor

## Abstract

Multiple endocrine neoplasia type 1 (MEN1) is a rare hereditary tumor syndrome inherited in an autosomal dominant manner and characterized by a predisposition to a multitude of endocrine neoplasms primarily of parathyroid, enteropancreatic, and anterior pituitary origin, as well as nonendocrine neoplasms. Other endocrine tumors in MEN1 include foregut carcinoid tumors, adrenocortical tumors, and rarely pheochromocytoma. Nonendocrine manifestations include meningiomas and ependymomas, lipomas, angiofibromas, collagenomas, and leiomyomas. MEN1 is caused by inactivating mutations of the tumor suppressor gene *MEN1* which encodes the protein menin. This syndrome can affect all age groups, with 17% of patients developing MEN1-associated tumors before 21 years of age. Despite advances in the diagnosis and treatment of MEN1-associated tumors, patients with MEN1 continue to have decreased life expectancy primarily due to malignant neuroendocrine tumors. The most recent clinical practice guidelines for MEN1, published in 2012, highlight the need for early genetic and clinical diagnosis of MEN1 and recommend an intensive surveillance approach for both patients with this syndrome and asymptomatic carriers starting at the age of 5 years with the goal of timely detection and management of MEN1-associated neoplasms and ultimately decreased disease-specific morbidity and mortality. Unfortunately, there is no clear genotype-phenotype correlation and individual mutation-dependent surveillance is not possible currently.

## Introduction

Multiple endocrine neoplasia type 1 (MEN1) is a rare autosomal dominant hereditary tumor syndrome with a high degree of penetrance, that is caused by inactivating mutations of the tumor suppressor gene *MEN1*, and is characterized by a predisposition to a multitude of endocrine and nonendocrine tumors (1). MEN1 was first described as early as 1903 by Erdheim and was defined by Underdahl and Werner ~5 decades later (2–4). The syndrome is classically comprised of hyperplasia and/or tumors of parathyroid, enteropancreatic, and/or anterior pituitary origin, that develop in 90, 30–70, and 30–40% of patients, respectively, by age 40 (5–8). Other endocrine tumors noted with increased frequency in MEN1 include foregut carcinoid tumors, such as thymic and bronchial carcinoid, and gastric enterochromaffin-like tumors, which each have a penetrance of 2%, 2%, and 10%, respectively by 40 years of age. Adrenocortical tumors develop in 40% of affected individuals by this age, whereas pheochromocytomas are rare (<1%). Nonendocrine manifestations are predominantly comprised of neoplasms of the central nervous system including, meningiomas, ependymomas, and schwannomas, cutaneous lesions such as lipomas, angiofibromas, and collagenomas, and smooth muscle tumors such as leiomyomas (9–15). Women with MEN1 appear to have an increased risk of breast cancer (16). MEN1 can affect all age groups with an estimated prevalence of 2 per 100,000 and no apparent gender predilection (17). Though extensive data is lacking regarding MEN1 in young individuals, in a cohort of 924 patients with MEN1, 160 patients, or 17%, developed MEN1-associated tumors before 21 years of age. In these young patients, primary hyperparathyroidism (PHPT) was diagnosed in 75% by age 21, followed by pituitary adenomas in 34%, pancreatic neuroendocrine tumors (pNETs) in 34% (14% non-functional pNETs [NF-pNETs], 12% insulinomas, and 2% gastrinomas), adrenal tumors in 1% (2 patients), and thymic neuroendocrine tumor (th-NET) in 1% (1 patient). MEN1-associated neoplasms were diagnosed as early as within the first 5 years of life, though the majority were diagnosed after the age of 10 with increasing disease penetrance with age (18).

Despite advances in the diagnosis and treatment of MEN1-associated tumors, patients with this syndrome continue to have a decreased life expectancy compared to the rest of the population, with a mean age of death of 55–60 years (19, 20). Furthermore, delayed MEN1 diagnosis has been associated with potential harm to patients, as demonstrated in a recent study where family members of MEN1 index cases developed metastatic NETs before or during the lag time (median lag time 3.5 years) between the diagnosis of MEN1 in the index case and genetic testing of family members for the disease (21, 22). The cause of death in 50–70% of patients with MEN1 is related to the disease itself (23–26). Through the advent of new treatments, the most common cause of death has shifted from the complications of hormone-excess states, primarily due to gastrinomas, to malignant NETs, most notably pNETs and th-NETs. The most recent Endocrine Society clinical practice guidelines for MEN1 published in 2012, recommend an intensive surveillance approach for patients with MEN1 and asymptomatic carriers starting at the age of 5 years, based on the assumption that early detection and management of MEN1-associated neoplasms may lead to decreased morbidity and mortality (8).

## Genetics and Molecular Pathogenesis

MEN1 may be inherited in an autosomal dominant manner, with 90% of individuals diagnosed with this disease having an affected parent, and only 10% having a *de novo MEN1* germline mutation (27). The *MEN1* gene, located on chromosome 11 (11q13), was first identified in 1997, and spans ~9,000 base pairs of genomic DNA containing 10 exons. This gene encodes the protein menin (1, 28). Germline heterozygous mutations in *MEN1*, are identified in 70–90% of familial MEN1 cases with the frequency of finding a *de novo* mutation being significantly lower in sporadic MEN1 cases (27). More than 1,200 germline mutations in the *MEN1* gene have been identified, which are scattered over the entire coding region of the gene without any significant hot spots or genotype-phenotype correlations (27, 29). The majority of *MEN1* germline mutations (69%) are predicted to be pathogenic due to either premature truncation of menin due to frame-shift mutations (42%) and nonsense mutations (14%), or exon region deletions which are attributed to splicing defects (10.5%) and large deletions (2.5%) (27, 29). Other *MEN1* germline mutations include missense mutations (25.5%) and single or few amino acid in-frame deletions or insertions (5.5%), which require further investigation to determine their pathogenicity.

Approximately 5–25% of patients with MEN1 may not have mutations in the *MEN1* coding region. These individuals may have whole or partial gene deletions, and it has been postulated that mutations may also occur in the promoter or untranslated regions (27, 30, 31). In addition, the occurrence of phenocopies, or patients that develop disease manifestations typically associated with mutations in the *MEN1* gene but instead are due to another etiology, has been described in 5–10% of MEN1 kindreds (32–34). These phenocopies may occur in individuals with a family history of MEN1 and one MEN1-associated tumor or in patients with two MEN1-associated tumors with other gene involvement.

MEN1 phenocopies can be attributed to multiple endocrine neoplasia type 4 (MEN4) in 1–2% of cases. This syndrome results from inactivating mutations of the tumor suppressor gene *CDKNIB*, that encodes the p27^kip1^ inhibitor of cyclin dependent kinase 2, with manifestations such as parathyroid and pituitary adenomas, neuroendocrine tumors and various benign, and malignant tumors. Less common germline mutations that may be identified in another 1–2% of phenocopies include those attributed to mutations in genes encoding additional members of the cyclin-dependent kinase inhibitor (CDKN) family, such as *CDKN1A* (P21^cip1^)*, CDKN2B* (p15^Ink4b^), or *CDKN2C* (p18^Ink4c^) (34–37). These CDKN genetic defects should be evaluated in patients that present as MEN1-like phenocopies.

Additional genes to be considered for screening in phenocopies include *CDC73* (also known as *HRPT2*) which is a tumor suppressor gene that encodes the protein parafibromin (mutations implicated in hyperparathyroid-jaw tumor syndrome), *CaSR* which encodes the calcium sensing receptor (mutations associated with familial benign hypocalciuric hypercalcemias), *GNA11* that encodes the G-protein alpha 11, and *AP2S1* which encodes the adaptor-related protein complex 2, sigma 1 subunit, particularly in patients with familial hyperparathyroidism. Defects in *AIP*, which encodes the aryl hydrocarbon receptor interacting protein and is associated with familial isolated pituitary adenomas, should also be evaluated in children and adolescents with prolactinoma or somatotropinoma (8). Germline *MEN1* mutations have also been noted in families with a parathyroid only disorder, familial isolated primary hyperparathyroidism, where there is a higher frequency of missense mutations compared to patients with the MEN1 syndrome (27, 29, 38, 39). Similarly, germline *MEN1* mutations have been reported in 5 cases of “sporadic” pNETs (40).

*MEN1* acts as a tumor suppressor gene. Patients with germline inactivating mutations in *MEN1* demonstrate loss of heterozygosity (LOH) in more than 90% of their tumors, though LOH involving chromosome 11q13 has also been observed in 5–50% of sporadic endocrine tumors (27). Neoplasms develop (as described in Knudson's two-hit hypothesis), when a second somatic inactivating mutation occurs in one *MEN1* allele in the setting of the preexisting germline inactivating mutation in the alternate allele (41). The protein product of *MEN1*, menin, is implicated in the regulation of transcription, genome stability, cell division, and cell proliferation, though the exact role of menin in tumorigenesis is yet to be elucidated (1, 27, 28, 42). Menin is composed of 610 amino acids, and is a highly conserved, ubiquitously expressed, predominantly nuclear protein, that does not show homology to any other protein. Two main nuclear localization signals (NLS), NLS1 and NLS2, as well as a third accessory NLS, NLSa, are harbored within the amino acid sequence of menin (43, 44). Mutations in *MEN1* that lead to premature protein truncation may lead to functional inactivation of menin through loss of one or both main NLSs. Menin has not been demonstrated to have intrinsic enzymatic activity, but studies of protein-protein interaction by multiple groups have identified more than 50 proteins that could partner with menin. Furthermore, the crystal structure of menin demonstrates a deep pocket that can serve as a binding site for interacting proteins (45–47). Menin is predicted to be a multi-functional protein that plays a role in epigenetic regulation and gene transcription through interaction with proteins in chromatin-associated protein complexes and transcription factors, with regulation of gene expression of target genes such as those that control cell proliferation. Similarly, through its protein partners, menin has also been implicated in playing a possible role in DNA-repair associated with response to DNA damage, cell signaling, cytoskeletal structure, cell division, cell adhesion, and/or cell motility (42, 48–50). In genetically engineered mouse models, germline targeted deletion of both copies of the *Men1* gene leads to death *in utero*, whereas germline targeted deletion of one copy of the *Men1* gene results in live mice that develop endocrine tumors similar to those in humans (47, 51).

Screening for *MEN1* mutations in the appropriate setting has several benefits including confirmation of the clinical diagnosis of MEN1, identification of family members that are carriers of *MEN1* mutations, so that appropriate screening and/or treatment can be implemented, and identification of the 50% of family members that do not harbor a pathogenic mutation in *MEN1* and thus do not require screening. Genetic testing for defects in the *MEN1* gene currently includes PCR-based screening for mutations in the coding region and splice junctions. If a mutation is not identified by the aforementioned method, then multiplex ligation probe amplification (MLPA)-based screening is performed in order to detect large deletions of the *MEN1* gene. This testing should be performed in a clinical genetics laboratory that is accredited to perform *MEN1* mutational analysis. Twenty-four different polymorphisms of the *MEN1* gene have been described and should be distinguished from mutations (27). According to the most recent clinical practice guidelines published in 2012 by Thakker et al. (8) *MEN1* mutational analysis should be performed in index cases with two or more MEN1-associated endocrine tumors (parathyroid, pancreatic, or pituitary tumors), asymptomatic first-degree relatives of a known *MEN1* mutation carrier, first-degree relatives of a *MEN1* mutation carrier expressing familial MEN1 (in order to exclude phenocopies), and in patients with suspicious or atypical MEN1 (individuals with parathyroid adenomas occurring prior to 30 years of age or multi-gland parathyroid disease, gastrinoma, or multiple pNET at any age, or those who have at least two MEN1-associated tumors not part of the classical triad of parathyroid, entero-pancreatic, and anterior pituitary tumors) (Table 1). When possible, this testing should be undertaken in the first decade of life, and as early as before the age of 5, with the goal of detecting and preventing significant morbidity and even mortality (8, 18, 52). All individuals should be provided genetic counseling prior to and following testing.

**Table 1:** Indications for MEN1 mutational analysis^*^ (8).

1. Index case with two or more MEN1-associated endocrine tumors (*i.e.*, parathyroid adenoma, enteropancreatic tumor, and pituitary adenoma) 2. Patients with suspicious or atypical MEN1, which includes individuals with parathyroid adenomas occurring before the age of 30; or multigland parathyroid disease, gastrinoma, or multiple pancreatic neuroendocrine tumors at any age; or individuals who have two or more MEN1-associated tumors that are not part of the classical triad of parathyroid, pancreatic islet, and anterior pituitary tumors 3.Asymptomatic first-degree relatives of a known *MEN1* mutation carrier 4. First-degree relative of a *MEN1* mutation carrier expressing familial MEN1 (*i.e.*, having symptoms, signs, biochemical, and/or radiological evidence for one or more MEN1-associated tumors)

*MEN1, multiple endocrine neoplasia type 1*.

* *MEN1 mutational analysis should be undertaken as early as possible (e.g., before the age of 5 for asymptomatic individuals); however, there is no clear genotype-phenotype correlation and surveillance should follow guidelines for all patients with documented MEN1*.

## Clinical Manifestations and Diagnosis

The diagnosis of MEN1 can be made on the basis of clinical, familial, and/or genetic criteria (Table 2). Per the most recent clinical practice guidelines, a patient can be diagnosed with MEN1 by meeting any one of the following three conditions: the occurrence of at least two primary MEN1-associated endocrine tumors (*i.e.*, parathyroid adenoma, enteropancreatic tumor, and pituitary adenoma); the development of one MEN1-associated tumor in a first degree relative of a patient with a clinical diagnosis of MEN1; and the identification of a germline *MEN1* mutation in an individual, who may be asymptomatic without biochemical or radiological evidence of MEN1 (8). Genetic evaluation in family members of patients with MEN1 was recently shown to result in the diagnosis of MEN 1, 10 years earlier than clinical or biochemical diagnosis (53).

**Table 2:** Diagnostic criteria for MEN1^*^ (8).

1. Occurrence of two or more primary MEN1-associated endocrine tumors (*i.e.*, parathyroid adenoma, enteropancreatic tumor, and pituitary adenoma) 2. Occurrence of one of the MEN1-associated tumors in a first-degree relative of a patient with a clinical diagnosis of MEN 3. Identification of a germline *MEN1* mutation in an individual who may be asymptomatic and has not yet developed serum biochemical or radiological abnormities indicative of tumor development

*MEN1, multiple endocrine neoplasia type 1*.

* *To make a diagnosis of MEN1, a patient must fulfill at least one of three criteria*.

### Parathyroid Tumors

Primary hyperparathyroidism (PHPT), due to parathyroid hyperplasia and/or adenoma, is the most common and often earliest endocrine manifestation in MEN1 and occurs primarily in the third decade of life with 100% penetrance by the age of 50 (5, 8). Though PHPT in children is predominantly diagnosed after the age of 10, asymptomatic PHPT has been described in patients with MEN1 as young as 4 years-old, with symptomatic disease diagnosed as early as 8 years of age (18). Furthermore, it is estimated that 1–18% of patients diagnosed with PHPT will have MEN1. The degree of hypercalcemia in these individuals is usually mild and patients may be asymptomatic or present with polydipsia, polyuria, constipation, malaise, altered mentation, hypertension, shortened QT interval, peptic ulcer disease, urolithiasis, and/or decreased bone mineral density with increased fracture risk. There is a high prevalence of urolithiasis and early bone mineral loss in young individuals with MEN1-associated PHPT, and bone or renal complications are progressively more frequent, extensive, and severe in long-standing PHPT cases and in those associated with gastrinoma (54–58). Severe hypercalcemia or parathyroid cancer are rare (59). Compared to sporadic PHPT, parathyroid disease in MEN1 occurs at an earlier age (20–25 years of age vs. 55 years) with an equal male to female ratio (1:1 vs. 1:3), and typically has multiple gland involvement, with ultimate involvement of all four glands (as compared to 80–85% of patients with sporadic PHPT that have single gland disease). MEN1-associated PHPT has a high recurrence rate after apparently successful subtotal parathyroidectomy that reaches up to 50% 12 years postoperatively (compared to 4–16% recurrence in sporadic PHPT) (60). In addition, a greater decline in bone mineral density is noted in patients with PHPT due to MEN1 when compared to patients with sporadic PHPT, with decreased bone mineral density recovery in the lumbar spine, femur, and particularly in the distal radius when compared to sporadic cases 1 year postoperatively (8, 60–63).

Parathyroid tumors in patients with multi-gland disease are asynchronous and vary in size, with each tumor likely representing a different clonal adenoma (64). In addition, a “negative feedback loop” between miR-24-1 and menin, has recently been suggested in MEN1-associated parathyroid tumorigenesis. This “negative feedback loop” mimics the second hit in Knudson's hypothesis by silencing the expression of the second *MEN1* wild type allele through a post-transcriptional, reversible, epigenetic effect, which may precede the permanent genetic deletion or inactivation of the second wild type allele (65).

PHPT is often diagnosed incidentally by biochemical testing that demonstrates hypercalcemia in association with inappropriately increased parathyroid hormone (PTH) levels. Given the multi-glandular nature of parathyroid involvement in MEN1, preoperative imaging to localize the parathyroid tumors, such as ultrasound of the neck, Tc99m-sestamibi parathyroid scintigraphy, or chest computed tomography (CT), is of limited value, with the exception of possible use in cases of recurrent or persistent PHPT. However, some groups perform pre-operative imaging in order to evaluate for ectopic parathyroid glands, or more recently, in order to plan for less extensive surgeries, but these protocols need to be validated by large series and post-operative long-term follow-up (66–69). Bilateral neck exploration during surgery should be planned in an attempt to identify all pathological parathyroid glands (70).

Surgery is the treatment of choice for PHPT though the optimal timing, type, and extent of parathyroid surgery are unclear and should be decided on a case by case basis. Whether early parathyroid surgery reduces morbidity and mortality in patients with MEN1 is yet to be elucidated. Some clinicians favor early intervention to reduce the years a patient is at risk of developing bone disease, while others prefer delaying surgery as much as possible with the goal of avoiding the risk of postsurgical hypoparathyroidism and limiting the number of surgeries a patient may need to undergo for management of recurrent PHPT. Indications for parathyroidectomy in patients with MEN1 are similar to those for individuals with sporadic PHPT and include symptomatic or marked hypercalcemia/hypercalciuria, nephrolithiasis, and/or evidence of bone disease (8, 71–74). Furthermore, parathyroidectomy may be required in the treatment of patients with severe peptic ulcer disease or other symptoms caused by gastrinoma (the Zollinger-Ellison syndrome [ZES]) that are poorly controlled with medical management, as hypercalcemia typically worsens hypergastrinemia (57, 75). The recommended surgical approach for the treatment of MEN1-associated PHPT is subtotal parathyroidectomy with removal of 3.5 parathyroid glands. Total parathyroidectomy with heterotopic auto-transplantation of fresh or cryopreserved normal parathyroid tissue into the forearm or neck may also be an option for certain patients, particularly those that have extensive disease either at initial or repeat surgery (8). Previous observational studies have noted that the total parathyroidectomy approach has been associated with lower rates of persistent hyperparathyroidism but higher rates of hypoparathyroidism. This was demonstrated in a review of 18 reports that included 2–73 patients with MEN1 followed for 4–12 years after subtotal parathyroidectomy with or without cervical thymectomy, where persistent hyperparathyroidism was noted in 0–33%, recurrent hyperparathyroidism in 0–36%, and persistent hypoparathyroidism in 0–35% of patients. The same review analyzed 10 reports that included 4–36 patients with MEN1 who underwent total parathyroidectomy with cervical thymectomy and heterotopic auto-transplantation of a small parathyroid graft, where after a mean follow up of 6–10 years, patients were noted to have persistent hyperparathyroidism in 0–3%, recurrent hyperparathyroidism in 0–55%, and hypoparathyroidism in 0–46% (76). Furthermore, in prior observational studies, patients who underwent less than subtotal parathyroidectomy more commonly had persistent disease, with 42% of patients who underwent <3 gland parathyroidectomy having persistent disease compared to 0–12% of those who had three or more parathyroid glands resected (57, 76–78). However, a recent retrospective study from a single institution that compared 8 patients with MEN1 that underwent “unilateral clearance” (preoperative localization of a single enlarged parathyroid gland with resection of that parathyroid gland, the remaining ipsilateral parathyroid gland, as well as ipsilateral transcervical thymectomy) to 16 patients with MEN1 who underwent subtotal parathyroidectomy, showed that one patient from each group had persistent disease (12.5 and 6.25%, respectively), 13% of patients with unilateral clearance and 31% of patients with subtotal parathyroidectomy had recurrent PHPT after a mean follow up of 47 (2–112) and 68 (1–223) months, whereas no unilateral clearance patients and 2 (12.5%) patients who underwent subtotal parathyroidectomy had permanent hypoparathyroidism (69). During parathyroidectomy, intraoperative monitory of PTH is suggested in order to monitor the correct ablation of all adenomatous and/or hyperplastic parathyroid glands (79). Furthermore, transcervical thymectomy is recommended in all patients with MEN1 during initial parathyroid surgery, as supernumerary glands are identified in 6–20% of MEN1 patients, and intrathymic parathyroid tissue may be a source of PTH excess (80, 81). Calcimimetics, such as cinacalcet, have been used to treat patients with hyperparathyroidism in whom surgery has failed or who were poor surgical candidates (82). Though data is limited in patients with MEN1, cinacalcet has been shown to have calcium lowering effects, but current evidence does not show an improvement in bone mineral density in the spine or femur or in hypercalciuria (83).

### Enteropancreatic Neuroendocrine Tumors

Pancreatic neuroendocrine tumors (pNETs) are estimated to occur in 30–80% of patients with MEN1, and in up to 80–100% of patients in postmortem studies. MEN1 is the most common hereditary syndrome associated with pNETs with approximately 10% of all pNETs being associated with MEN1 (84). In addition, somatic *MEN1* mutations can be observed in >40% of sporadic pNETs (40, 85). These tumors may secrete excessive quantities of hormone such as gastrin, insulin, vasoactive intestinal polypeptide (VIP), glucagon, or somatostatin and may be associated with distinct clinical syndromes, with approximately one third of these neoplasms becoming clinically apparent. Also, these tumors may be nonfunctional (NF-pNET), either non-secretory, or releasing hormonally inactive peptides such as pancreatic polypeptide, chromogranin A, neurotensin, neuron-specific enolase, or ghrelin (8). By the age of 40, the prevalence of gastrinoma is 30–40%, that of insulinoma is 10%, and that of other functioning pNETs such as glucagonoma, VIPoma, somatostatinoma, etc. is 2%, whereas the prevalence of non-functioning enteropancreatic tumors including those that secrete pancreatic polypeptide is 20–55% (5). Pancreatic neuroendocrine tumors have an earlier age of onset in patients with MEN1, when compared to sporadic cases (10–50 years of age *vs*. 50–80 years), and tend to be multiple (as opposed to single in sporadic pNETs), most commonly leading to diffuse microadenomatosis (tumors <0.5 cm), with <13% of patients developing larger tumors (>2 cm) which are often nonfunctional (5, 6, 86–88). As previously mentioned, given the effective treatment of PHPT, pituitary disease, and Zollinger-Ellison syndrome (ZES), the malignant potential of enteropancreatic NETs, particularly NF-pNETs, is now the primary cause of death in patients with MEN1. Several MEN1 genotype-phenotype correlations regarding pNET growth and/or malignant potential have been identified (mutations in JunD, CHES1, truncating mutations in the N- or C- terminal regions of the *MEN1* gene [exons 2,8,9] and the *CDKN1B* V109G polymorphism), and particular pathological characteristics such as high mitotic count in large NF-pNETs, may characterize more aggressive tumors, however these have not been studied prospectively and are not broadly used (7, 89–95).

Gastrinoma is the most common functional enteropancreatic tumor in MEN1 and may lead to ZES (hypergastrinemia with recurrent peptic ulcerations), with ZES being the initial clinical manifestation in up to 40% of patients with MEN1. Up to 60% of patients with MEN1 have either ZES or asymptomatic elevation in serum gastrin concentration (96). Furthermore, 20–60% of patients with ZES have underlying MEN1 (97). ZES may present with gastroesophageal reflux disease (GERD) and peptic ulcer disease (PUD), which are often refractory, with or without diarrhea. In a study of 160 patients with MEN1 below the age of 21, 6 patients (or 2%), developed ZES before the age of 21, with the youngest patient to develop the disease being 6 years of age (18). Gastrinomas in MEN1 are typically diagnosed by the age of 40 (on average 10 years earlier than sporadic gastrinomas) and are most commonly microadenomas that occur primarily in the duodenum (>80%), and less frequently in the pancreas. They may be complicated by lymph node metastases in 34–85% of cases, and hepatic metastases in 6–16% of cases at the time of diagnosis (98–100). The diagnosis of gastrinoma is indicated by elevated fasting serum gastrin concentration (gastrin levels 10 times greater than the upper limit of normal) in the presence of hyperchloridria or gastric pH <2. If fasting gastrin levels are below this threshold (1,000 pg/ml), then gastrin stimulation (either by secretin or calcium infusion) may be necessary for diagnosis. Localization, which should include evaluation for metastases, may be comprised of gastroscopy and endoscopic ultrasound (EUS), particularly for duodenal gastrinomas, as well as other imaging procedures (ultrasound [US], CT, magnetic resonance imaging [MRI], selective abdominal angiography, somatostatin receptor scintigraphy [with In-DPTA-octreotide or octreoscan], ^68^Ga-DOTATATE-PET [positron emission tomography]/CT), with combined EUS and MRI being the most sensitive for pancreatic disease. The combined use of intraarterial calcium injections with hepatic venous gastrin sampling may also be used for diagnosis (101–103). Though they typically grow slowly, gastrinomas can frequently metastasize to the peripancreatic lymph nodes and less commonly to the liver, with a higher mortality rate than insulinomas but not than other pNETs (26). Lymph node metastases do not inevitably result in a poor prognosis or a high probability that clinically significant metastases will occur, and the risk of death from lymph node metastases due to MEN1 associated gastrinoma is less than that for sporadic cases (104). The prognosis of patients with MEN1-associated gastrinomas is associated with tumor size and the presence of hepatic metastases, with increased risk of hepatic metastases with increasing tumor size (103, 105). Treatment of MEN1-associated gastrinomas is primarily comprised of medical therapy which aims to decrease gastric acid secretion through the use of histamine 2-receptor antagonists, proton pump inhibitors, and/or somatostatin analogs (SSAs). These medications have shown long-term effectiveness and safety in controlling hypergastrinemia and the complications of ZES. Furthermore, SSAs have been demonstrated to have an anti-neoplastic effect, though data is lacking regarding their effectiveness in malignant and/or metastatic gastrinoma (106, 107). Surgical treatment of gastrinomas/ZES in patients with MEN1 is controversial given the multiplicity and small size of these tumors, that leads to rare surgical cure without aggressive resections. Surgery is suggested when concomitant NF-NETs double in size over 6 months or exceed 2 centimeters in diameter, with possible surgical approaches including Thompson's procedure (excision of the duodenal gastrinoma through a longitudinal excision), duodeno-pancreatectomy, or pancreas-preserving surgery (8, 108). The use of surgical resection is also limited in advanced metastatic disease, with treatment involving several approaches that may include medical therapy (with everolimus, tyrosine kinase inhibitors such as sunitinib, or chemotherapy), peptide radioreceptor therapy with lutetium-177-labeled somatostatin analog, chemotherapy, or liver directed therapies (such as embolization, chemoembolization, or radioembolization) (109). As aforementioned, hypercalcemia from concomitant PHPT may worsen the symptoms of ZES, with parathyroidectomy leading to decreased fasting and secretin simulated gastrin levels and basal acid secretion (57). Another special consideration in patients with ZES is the increased incidence of Cushing syndrome that has been described in these patients, which is due to a corticotroph pituitary adenoma causing Cushing disease, as opposed to ectopic ACTH secretion from an islet cell tumor which is more commonly seen in conjunction with sporadic gastrinomas (110).

Insulinomas are the second most common type of functional enteropancreatic neoplasm in patients with MEN1, representing 10–30% of pNETs in this group (8). They are the most frequent cause of endogenous hyperinsulinemic hypoglycemia in adult patients, with approximately 4% of insulinomas occurring in patients with MEN1. These tumors comprise the first MEN1-associated manifestation in 10% of patients with the syndrome. In contrast to sporadic insulinomas, which tend to occur after the age of 40, MEN1-associated insulinomas occur primarily in patients younger than 40 years-old, with many of these tumors arising in patients younger than 20 years of age (5, 17, 18, 86, 111). In the aforementioned cohort of 160 patients with MEN1 below the age of 21, insulinoma occurred in 10% of patients and as early as 5 years of age (18). These tumors are solitary in 85% and multiple in 6–13% of cases, and are associated with other pNETs at the time of diagnosis in 10% of patients (112). They are scattered across the pancreas and tend to be small, with 82% of these neoplasms measuring less than 2 centimeters and 47% measuring less than 1 centimeter, which may make them difficult to localize (113). Diagnosis is established with a 72-hour fast through documentation of hypoglycemia with characteristic symptoms that are rapidly reversed by glucose administration and with demonstration of inappropriately increased plasma insulin, c-peptide, and/or proinsulin, in the setting of hypoglycemia (8). Localization is pursued with ultrasound, CT, and/or MRI, with EUS demonstrating a sensitivity of 94% (114). If these studies fail to localize the insulinoma(s), more invasive techniques such as selective angiography with intra-arterial calcium stimulation and hepatic venous sampling for insulin levels may localize more than 80% of these neoplasms (115, 116). In addition, more novel somatostatin receptor-targeting PET modalities such as ^68^Ga-DOTATATE-PET/CT, may aide in localization, with PET imaging with labeled glucagon-like peptide 1 (GLP1) analogs being a promising possible future modality (117–120). Medical therapy is often unsuccessful in patients with MEN1-associated insulinomas, with surgery being the treatment of choice. Unlike patients with MEN1-associated gastrinoma/ZES that are rarely cured with pNET enucleation or local gastrinoma excision, and often require more extensive resections, those with other functional pNETs including insulinomas are often cured without extensive surgical resections, but may have recurrent disease (108). There is no consensus on the optimal extent of surgical resection in patients with insulinoma, which can range from enucleation of a single tumor, to distal pancreatectomy or partial pancreatectomy, to excision of all the macroscopic pancreatic neoplasms and enucleation of nodules in the remaining pancreas (8). Monitoring of the insulin/glucose ratio during surgery may be helpful in assessing successful insulinoma excision (121). Many experts suggest distal pancreatic resection associated with enucleation of any additional pancreatic head tumors as the treatment of choice (122, 123). Metastatic insulinoma is rare, occurring in 4–14% of cases, with the goal of treatment, as in other malignant functioning pNETs, being symptom control and tumor volume reduction with possible resection of lesions that are amenable to surgery (124, 125). In cases with unresectable disease or in patients that need further symptom control, adjuvant therapy may vary from diazoxide and SSAs to everolimus and chemotherapy, as well as local therapies (radiofrequency ablation [RFA], chemoembolization, or radiotherapy) or peptide receptor radionuclide therapy (PRRT) (112).

Glucagonomas occur in <3% of MEN1 patients, though NF-pNETs may have positive immunostaining for glucagon (5, 111, 121, 126). These tumors most frequently occur in the tail of the pancreas, and 50–80% of patients have metastases at the time of diagnosis (127). Patients may present with characteristic symptoms such as skin rash (necrolytic migratory erythema), weight loss, anemia, and stomatitis (8). Asymptomatic patients may be detected incidentally through findings on imaging and/or biochemical evidence of glucose intolerance or hyperglucagonemia. VIP-secreting tumors or VIPomas, are rare in MEN1 and present with watery diarrhea, hypokalemia, and achlorhydria (WDHA) (127). Like glucagonomas, these neoplasms are primarily located in the pancreatic tail. The diagnosis of a VIPoma is made through evidence of a fasting stool volume >1–2 liters per day and a significantly increased plasma VIP concentration, in the absence of laxative or diuretic use (8). The treatment approach for both glucagonomas and VIPomas is similar to that for insulinomas as described above (112).

Non-functioning pNETs are among the most common enteropancreatic tumors in MEN1 (6, 7, 128, 129). Detection of NF-pNETs has significantly increased in the setting of standardized biochemical and imaging protocols in patients with MEN1 (8, 128, 130). Furthermore, pathology from pancreatic resections in patients with MEN1 has revealed numerous microtumors that were not shown preoperatively on imaging (131). NF-pNETs have been detected in patients with MEN1 as early as 12–14 years of age, and patients between the ages of 10 and 20 have been reported as developing large tumors (18, 87, 132, 133). EUS is the most sensitive modality for detecting NF-pNETS, with a combined strategy of EUS and MRI being the most useful. ^68^Ga-DOTATATE-PET/CT has been reported to have a high sensitivity for detecting NETs in MEN1, at times leading to a change in management. This study may be added to identify metastases in patients with NF-pNETs (119, 134). In addition, ^18^F-fluorodeoxyglucose (FDG) PET/CT imaging can be valuable in predicting the malignant potential of pNETs (135). There is lack of agreement regarding the optimal indications for surgery in patients with NF-pNETs, with major considerations including the size of the neoplasm and other considerations being the growth rate, presence of metastases, and tumor histology. Previous studies have shown an increased rate of metastases in patients with larger tumors (6, 7, 87, 136). However, there is controversy among experts regarding the tumor size cutoff for offering surgery, with the suggested cutoff ranging between 1 and 3 centimeters (8, 112, 137). There is a consensus that withholding surgery for tumors that reach or exceed 3 centimeters in size leads to a negative prognosis, however the indication for surgical intervention in smaller-sized tumors is unclear (138). The Endocrine Society clinical practice guidelines for MEN1 suggest surgical resection for NF-pNETs >1 cm in size, or tumors <1 cm in size with significant growth, such as doubling of tumor size over 3–6 months and exceeding 1 cm in size (8). The 1-centimeter cutoff for surgical NF-pNET resection has also been suggested by several other groups of experts including the Uppsala group, the Marburg group, and the MEN consortium in Japan (139–141). However, the European Neuroendocrine Tumor Society and the Groupe d'Etude des Tumeurs Endocrines (GTE) suggest conservative management for tumors <2 cm in size that do not demonstrate characteristics of aggressive behavior such as rapid growth (108, 136). Furthermore, two recent studies advocating for conservative management for NF-pNETs that are 2 cm or less in size, demonstrated low disease-specific mortality without loss of oncologic safety at this threshold (138, 142). In terms of surgical approach, the least invasive procedure is typically considered best, with lymph node dissection in addition to pancreatic resection according to the type and location of the tumors (108). Treatment options for unresectable pNETs or advanced metastatic disease may include biotherapies such as SSAs, interferon alpha, mechanistic target of rapamycin (mTOR) inhibitors, receptor tyrosine kinase (RTK) inhibitors, including platelet-derived growth factor receptor (PDGFR) and vascular endothelial growth factor receptor (VEGFR) inhibitors, and vascular endothelial growth factor A (VEGFA) antibodies. Chemotherapy may be appropriate for patients with metastatic pNETs with a high tumor burden, high proliferative index, rapid tumor progression, and/or symptoms not controlled with biotherapy. Radiological therapies such as PRRT, or localized interventional radiological treatment using RFA, transarterial embolization (TAE), transarterial chemoembolization (TACE) and selective internal radiation therapy (SIRT) may also be considered in select cases (143).

### Anterior Pituitary Tumors

The prevalence of anterior pituitary tumors in patients with MEN1 varies widely among series between 10 and 60%, and was noted to be 42% and 38.1% in two large cohorts (144–146). Pituitary tumors (PITs) have been reported as early as the age of 5 years and can occur as late as the ninth decade of life, with onset primarily in the fourth decade (30, 52). Furthermore, these tumors were noted in 35% of patients in a large cohort of pediatric patients, comprising the second most common MEN1-associated neoplasm in this group, and the first lesion identified in 21% of cases (18). Overall, PITs may be the first MEN1-associated lesion to develop in at least 10–15% of cases, and up to 3% of patients with the diagnosis of a PIT will have MEN1 (111, 147–149). Approximately 60% of these tumors secrete prolactin (PRL), 25% release growth hormone (GH), 5% secrete ACTH, and the remaining are primarily nonfunctional (NFTs) (147, 150, 151). This profile is similar to that of sporadic PITs but differs from that of other hereditary pituitary tumors (due to *GNAS, AIP*, or *PRKAR1A* defects) that secrete primarily GH. In a study that included 324 patients, MEN1-associated PITs were described as being larger (macroadenomas in 85 vs. 42% in sporadic cases), with more aggressive behavior (one third of MEN1-associated cases were observed to have invasive features), and reduced response to medical therapy (42 vs. 90% in sporadic cases), but without increased prevalence of pituitary carcinoma (146, 147, 151). However, in a subsequent study that included 323 patients with MEN1, two thirds of the PITs were microadenomas. Tumors detected by screening were predominantly nonfunctional microadenomas, that did not require treatment and were stable in size over time. Symptom development that required treatment was rare during long-term follow-up (median 6 years). Furthermore, the response rate to medical therapy of prolactinomas was more than 90% (144). MEN1-associated PITs have been shown to demonstrate plurihormonal expression more frequently than sporadic tumors (146, 151). However, in contrast with other MEN1-associated neoplasms, multiple pituitary tumors are rare (152). Clinical manifestations of PITs in MEN1 are similar to those with sporadic PITs, and include those that are due to hormone excess, and those that are related to the size of the tumor. Patients with prolactinomas may present with amenorrhea, galactorrhea and infertility in women, or erectile dysfunction and infertility in men. Those with excess GH or ACTH secretion may present with signs or symptoms of gigantism or acromegaly, or Cushing syndrome, respectively. Individuals with enlarging PITs may experience headaches and signs of compression of adjacent structures such visual field disturbances in the setting of optic chiasm compression, and/or hypopituitarism. Biochemical evaluation can lead to the diagnosis of hormone excess syndromes, whereas MRI of the pituitary gland is the imaging study of choice to diagnose PITs (8). Treatment of PITs in patients with MEN1 is similar to treatment in sporadic disease. Medical therapy with dopamine agonists is first-line for prolactinomas, whereas transsphenoidal surgery (TSS) and radiotherapy are reserved for drug-resistant tumors. For GH- and ACTH-secreting tumors the initial treatment of choice is often TSS. When surgery is indicated, TSS is also the primary treatment for non-functioning PITs, particularly in macroadenomas associated with visual signs and symptoms or that are in close proximity to the optic chiasm and/or those that demonstrate rapid growth.

### Other Tumors

Several other neoplasms occur with increased frequency in MEN1 including foregut carcinoid tumors, adrenocortical tumors, cutaneous tumors, meningiomas and ependymomas, tumors of smooth muscle, and very rarely pheochromocytomas.

MEN1-associated carcinoid tumors include thymic, bronchial, and gastric enterochromaffin-like cell NETs (8). Thymic NETs (th-NETs), occur in 2–8% of patients with MEN1, and are associated with strong heritability and a poor prognosis with frequent recurrence and increased risk of death (153–159). They occur mostly in men, whereas carcinoids in women are primarily bronchial (157, 160, 161). Heavy smoking may be a risk factor (161). Th-NETs have been identified as early as 16 years of age and may be fatal even in young patients (18). These tumors are primarily nonfunctional and are the most common cause of anterior mediastinal masses in MEN1. Most individuals may be asymptomatic at diagnosis, however liver, bone, and lung metastases may be identified. Biochemical evaluation is not useful diagnostically in these patients with the diagnostic studies of choice being CT or MRI of the chest (112, 162). MEN1-associated th-NETs comprise the second most common MEN1-related cause of death and have a high mortality rate, with the 10-year survival rate in several larger studies ranging between 25 and 45% (153, 157, 163). Treatment is primarily surgical and complete resection of thymic tumors is recommended. Adjuvant radiotherapy may be used for incomplete resection and/or positive surgical margins. Medical treatments that can be considered for unresectable/metastatic disease, include those that are implemented in sporadic th-NETs such as SSAs, chemotherapy, mTOR inhibitors, and PRRT (112). Prophylactic transcervical thymectomy is typically performed at the time of parathyroidectomy for PHPT, however this does not eliminate the risk of development of th-NETs, due to residual thymic tissue (81, 164). The frequency of bronchial NETs (br-NETs) in MEN1 has been reported to range from 3 to 13%, and may even reach 31%, depending on the mode of diagnosis (imaging and/or pathology), with the frequency of histologically proven br-NETs ranging from 4.6 to 6.6% (153, 158, 165–168). Br-NETs have not been described in patients younger than 20 years old, with increasing penetrance with age which reaches 8.1% at 60 years (165). Presenting symptoms may include dyspnea, hemoptysis, cough, and/or flushing, however most patients are asymptomatic (158, 165, 169). Several cases of br-NETs have been described in the same families (165). Most br-NETs are typical or atypical carcinoids, though small- and large-cell neuroendocrine carcinomas were identified in 10% of cases in a cohort of 51 patients (165). Alhough, br-NETs do not decrease overall survival in patients with MEN1, poorly differentiated and aggressive br-NETs can lead to death. Chest CT or MRI may be used to detect these tumors. Management is similar to that for sporadic br-NETs, with surgical resection being the recommended treatment modality, though it is possible that small non-central lesions can be followed without affecting mortality (112). Gastric carcinoids (type II gastric enterochromaffin-like cell [ECL] carcinoids) and ECL proliferation (a precursor to gastric carcinoid) have been described in patients with MEN1 and ZES. In a cohort of 57 patients with MEN1 and ZES, ECL proliferative changes were universally present, whereas advanced changes were noted in 53% and carcinoids were diagnosed in 23% (170). Recent studies have demonstrated that these Type II gastric carcinoids may be aggressive and metastasize to the liver. However, disease is localized in at least 70 to 90% of cases and these tumors can often be excised endoscopically after assessment of the extent of invasion by EUS. In certain patients, tumors are present in excessive numbers or are large and may be invasive. In these patients, more aggressive resection and additional therapy with SSAs or CCK-B receptor antagonists may be considered (171). Annual surveillance with endoscopy is recommended.

Asymptomatic adrenocortical tumors (ACTs) may be detected in 20–73% of patients with MEN1 depending on the imaging modality used for screening (172–175). These lesions are primarily nonfunctioning and may include cortical adenomas that may be multiple, hyperplasia, cysts, or carcinomas. Less than 10% of MEN1-associated ACTs demonstrate hormone hypersecretion, which can lead most commonly to primary hyperaldosteronism and/or ACTH-independent Cushing syndrome (174). Hyperandrogenemia may occasionally be detected, particularly in the setting of an adrenocortical carcinoma (ACC). ACC has been described in 1% of patients, manifesting as early as 3 years of age. In the previously described group of 160 young patients under 21 years of age, 2 patients were diagnosed with ACC (18). Pheochromocytoma is rare with a prevalence of <1% by age 40 (5). Patients with signs or symptoms concerning for a functional ACT or pheochromocytoma, and those with tumors >1 cm on imaging should be evaluated biochemically for hormone excess. The incidence of ACC in tumors >1 cm in size rises to 13% (174). Patients with MEN1 and adrenal tumors should receive annual imaging, with consideration for surgical tumor resection if tumors are larger than 4 centimeters, display atypical radiological features and are 1–4 cm in size, or show significant growth over 6 months. Treatment of functional adrenal tumors in MEN1 is similar to that of sporadic neoplasms (172–174).

Cutaneous manifestations are common in MEN1 and may include lipomas, angiofibromas, and collagenomas, with the penetrance of these lesions by age 40 being 30, 85, and 70%, respectively (5). Angiofibromas and collagenomas tend to be multiple and are more common in patients with MEN1 than in the general population (64% *vs*. 8% and 62% *vs*. 5%, respectively). The presence of angiofibromas and/or collagenomas may be a strong indicator of the diagnosis of MEN1. In one study that included 110 patients with gastrinoma, including 48 individuals with MEN1 and 62 without MEN1, the presence angiofibromas or collagenomas (single or multiple), had a sensitivity of 50–65% for MEN1 and a specificity of 92–100%. The combined finding of at least 3 angiofibromas and any collagneoma had a sensitivity of 75% and a specificity of 95% in diagnosing MEN1 (176).

## Surveillance

The most recent Endocrine Society clinical practice guidelines for MEN1 recommend a comprehensive surveillance scheme commencing at the age of 5, with the goal of early detection and management of MEN1-associated manifestations and tumors (Table 3) (8). Unfortunately, there is no clear genotype-phenotype correlation and individual mutation-dependent surveillance is not possible at this time.

**Table 3:** Recommended biochemical and radiological surveillance for MEN1-associated tumors in patients with MEN1 and *MEN1* mutation carriers (8).

**Tumor**	**Age to begin screening (years)**	**Biochemical test (serum or plasma) annually**	**Imaging test (time interval)**
Parathyroid	8	Calcium, PTH	None
Gastrinoma	20	Gastrin (± gastric pH)	None
Insulinoma	5	Fasting glucose, insulin	None
Other pNETs	<10	CgA; PPP; glucagon; VIP	MRI, CT, or EUS (annually)
Anterior pituitary	5	Prolactin, IGF-1	MRI (every 3 years)
Adrenal	<10	None unless symptoms or signs of functioning tumor and/or tumor >1 cm identified on imaging	MRI or CT (annually with pancreatic imaging)
Thymic and bronchial carcinoid	15	None	CT or MRI (every 1–2 years)

*MEN1, multiple endocrine neoplasia type 1; PTH, parathyroid hormone; pNET, pancreatic neuroendocrine tumor; CgA, chromogranin A; PPP, pancreatic polypeptide; VIP, vasoactive intestinal peptide; MRI, magnetic resonance imaging; CT, computed tomography; EUS, endoscopic ultrasound; IGF-1, insulin like growth factor-1*.

Screening for hyperparathyroidism with annual biochemical evaluation of calcium and PTH is recommended starting at the age of 8, which is the age at which the earliest symptomatic patient with PHPT has been described (asymptomatic hyperparathyroidism has been observed as early as age 4) (18). Biochemical evaluation for gastrinoma is recommended beginning at the age of 20, though 2% of cases have been detected in patients younger than 21 years-old. Screening for gastrinoma is performed through annual evaluation of gastrin (± gastric pH). Evaluation for insulinoma should commence at age 5, which is the age at which the earliest symptomatic case has been reported, and should include annual insulin and glucose measurement. For other pNETs the annual measurement of chromogranin-A, pancreatic polypeptide, glucagon, and VIP is recommended beginning before the age of 10. However, the sensitivity of these measurements to detect foregut NETs is unclear, as two retrospective analyses of the use of chromogranin A, pancreatic polypeptide, and glucagon to screen for the emergence of MEN1-associated neoplasms found that these tests were not effective in early tumor diagnosis, either singly or in combination (177, 178). Imaging with MRI, CT, or EUS, is also recommended annually to evaluate for pNETs beginning before the age of 10 years, as large pancreatic tumors may develop between 10 and 20 years of age. Surveillance for anterior PITs is recommended starting at age 5, which is consistent with the age of the earliest case of MEN1-associated PIT that has been reported and includes annual measurement of serum PRL and insulin-like growth factor 1 (IGF-1) and MRI of the pituitary every 3 years. Evaluation for MEN1-associated adrenal tumors is primarily based on imaging either with CT or MRI that can be performed in conjunction with pancreatic imaging, with biochemical testing indicated only in the setting of symptoms or signs of a functional tumor or the presence of a tumor >1 cm. Screening for adrenal neoplasms is recommended beginning before the age of 10, as ACC has been identified in a child as young as 3 years of age. Lastly, screening for thymic and bronchial NETs is recommended starting at the age of 15, with CT or MRI recommended every 1–2 years. This is consistent with the diagnosis of fatal cases of th-NETs in patients as young as 16 years-old, though br-NETs have not been reported in patients younger than 20 years-old.

## Author Contributions

CS and CK wrote and contributed to the manuscript equally.

### Conflict of Interest Statement

CS holds patents on *PRKAR1A* and other genes of the cyclic AMP pathway and their genetics and applications. CS lab has received research funding from Pfizer Inc. for the study of gigantism and/or acromegaly. The remaining author declares that the research was conducted in the absence of any commercial or financial relationships that could be construed as a potential conflict of interest.
